# Global Health Innovation Technology Models

**DOI:** 10.5772/62921

**Published:** 2016-01-01

**Authors:** Kimberly Harding

**Affiliations:** 1 Monarch Innovation Partners, Inc, Rockville, USA

**Keywords:** Radiology, Imaging, Global Health, Low Income Countries, Low Middle Income Countries, mHealth, ehealth, Clinical Trials, Drug Discovery, CRO, Infectious Diseases, Rare Diseases, Tropical Diseases, TB, Public Health, Health Information Technology, Health Information Management, FDA, WHO, Open Science, Open Source: Big Data, Clinical, Informatics, Bioinformatics, Epidemiology, Medical Device, Software, Hardware, GCP, ICH, Africa, Tanzania, HIV, Malaria, Clinical Imaging, Nanotechnology, Nanomedicine, Innovation, Commercialization, Regulatory, Standards, Frameworks, Semantics, Syntax, Interoperability, Business Process, Product Development

## Abstract

Chronic technology and business process disparities between High Income, Low Middle Income and Low Income (HIC, LMIC, LIC) research collaborators directly prevent the growth of sustainable Global Health innovation for infectious and rare diseases. There is a need for an Open Source-Open Science Architecture Framework to bridge this divide. We are proposing such a framework for consideration by the Global Health community, by utilizing a hybrid approach of integrating agnostic Open Source technology and healthcare interoperability standards and Total Quality Management principles. We will validate this architecture framework through our programme called Project Orchid. Project Orchid is a conceptual Clinical Intelligence Exchange and Virtual Innovation platform utilizing this approach to support clinical innovation efforts for multi-national collaboration that can be locally sustainable for LIC and LMIC research cohorts. The goal is to enable LIC and LMIC research organizations to accelerate their clinical trial process maturity in the field of drug discovery, population health innovation initiatives and public domain knowledge networks. When sponsored, this concept will be tested by 12 confirmed clinical research and public health organizations in six countries. The potential impact of this platform is reduced drug discovery and public health innovation lag time and improved clinical trial interventions, due to reliable clinical intelligence and bio-surveillance across all phases of the clinical innovation process.

## 1. Introduction

### 1.1 Challenges Faced by Emerging Markets and Developing Countries in Global Health Innovation Efforts

LMICs and LICs have been impacted by chronic disparities in the adoption and advancement of clinical trial and population health innovation efforts, due to conflicting regulatory requirements for Phase I to Phase IV clinical trial models primarily defined in the Western world [1, 2]. An excerpt from the WHO Public Health Innovation report [3] summarizes this current dilemma: “*Scientists in developing countries should be involved in the development of the research protocol from the beginning to ensure that local health needs of developing countries are taken account of. Otherwise, the reality will be that physicians and researchers in developing countries who take part in conducting clinical trials are placed in the role of data collectors for trials designed only to fit the needs of people in the developed world. Measures and policies should be implemented to ensure that these physicians and researchers can design and initiate clinical trials that address health problems in their own countries, rather than fulfil research protocols designed elsewhere.*”

## 2. Examples of Impeded Progress in Emerging Markets: India

In the last two decades, India has become one of the most sought-after locations within the cluster of emerging markets for global clinical trials, due to its potential for fast recruitment of patients. However, India's ability to sustain its growth within the drug discovery domain continues to be significantly challenged by both regional and global regulatory changes [4]. According to Dr. Arun Bhatt, President of Clininvent Research Private Limited in Mumbai, India, the clinical trial protocols during this period of growth within India became more complex, demanding and inefficient for both the research teams and patients. Between 1999 and 2005 [4], the average number of inclusion criteria increased threefold. The average number of procedures grew annually by 6.5%, reaching a median number of 35 procedures in 2005. In 2012, a typical phase III protocol included 50 eligibility criteria, 167 procedures and 13 endpoints [4].

## 3. Gaps in Global Health Innovation

The root causes of these disparities are based on a lack of an agile technology infrastructure and robust ideation processes that can be adapted to their clinical research environments. Fragmented clinical trial processes and infrastructure deficiencies have left many promising research scientists with the inability to fully collaborate with their HIC peers. As a result, these researchers are unable to consistently partner on acute pandemic viral outbreaks, preventive medicine initiatives and new vaccine development that could save millions of lives. The International Council for Harmonisation (ICH) has identified the following unresolved areas of drug discovery innovation [5, 6], shown below in Table 1:

According to ICH, if these barriers are addressed, significant progress in the area of drug discovery and medical innovation can be achieved, resulting in [5, 6] a reduction in the costs of internal failures (rejects, reworks, reprocessing and investigations). This also includes optimized regulations to enable LMICs and LICs to meet drug discovery submission criteria and expedite the availability of medicines to patients. There are clear signs that the Healthcare and Life Science industries are ready to address these barriers, due to [7, 8] a growing demand for more focused research on infectious, rare, poverty-based and tropical diseases worldwide. In addition, there is a re-invigorated global movement towards clinical trial policy harmonization and acceptance of imported, de-identified clinical data sets for cross-trial analysis. Collectively, these changes in the Global Health Innovation community have resulted in the formation of new collaboration frameworks designed to bridge infrastructure and clinical intelligence disparities between HICs and LICs.

**Table 1.** ICH Barriers to Global Health Innovation

Unresolved Areas of Drug Discovery within the Global Health Innovation Community
Fragmented approaches to quality systems related to Good Clinical Practices (GCP) internationally
Suboptimal deployment of limited resources to identify, enact or support effective elements of a quality system and continual improvement by both industry and regulatory agencies
Delays may occur in the availability of medicines to patients round the world due to vast disparities in access to robust quality management systems and mature clinical trial practices
Delays in the implementation of innovation and continual improvement of existing products may occur due to differences in expectation across differing regulatory bodies around the world
Inability to implement consistently across stakeholder best practices related to Total Quality Management in other industries, which contributes to a lack of agility and repeatability in the quality of clinical trial practices

## 4. Pharmaceutical and Medical Innovation Model Approaches

In response to the change in the industry for expanded innovation worldwide, the International Federation of Pharmaceutical Manufacturers and Associations (IFPMA) has developed an innovation framework called the Pharmaceutical Innovation Platform (PIP). The focus of PIP is to provide a framework for a sustainable clinical innovation model, supported by collaborative partnerships, to achieve new heights in drug discoveries and healthcare solutions [9]. An excerpt from its PIP framework emphasizes this point [9]:

*“Healthcare, science and medicine challenges are global – all parties need to collaborate to meet these challenges effectively. Innovation is the vital element in this effort: when the public sector, industry, and civil society pull together to promote innovation, public health improves and lives are saved.”*

The IFPMA continues to state that PIP is not only achievable for industrialized countries, but for developing countries as well. “*Partnerships among established R&D companies, local R&D companies, international organizations, and local governments can be effective ways to harness the expertise of the various partners to find new treatments and cures for diseases which primarily affect poor countries* [9]”.

**Table 2.** IFPMA PIP Model Attributes

Tenets of IFPMA PIP Model	Healthcare Innovation and Delivery System Attributes
**Successful Healthcare Systems**	Efficient medical delivery and distribution systems Overall healthcare culture and policies promoting innovation Strong patients' groups Good access to pharmaceutical information
**Efficient Markets**	Healthcare expenditure seen as investment, not cost Realistic assessment of the pharmaceuticals in improving healthcare, including the real value of incremental innovation Efficient and transparent pricing and reimbursement in the decision-making process International price variations
**Effective Use of Intellectual Property**	Effective enforcement of intellectual property rights Sufficient and respected market exclusivity periods Prevention of parallel trade
**Adequate and Predictable Regulatory Requirements**	Stable and predictive regulatory environment Cooperation between regulators and industry Swift and transparent drug regulatory approval process Global harmonization of regulatory requirements Adjustment of regulatory requirements to advances in science and technology

**Table 3.** Summarized Collaborative Innovation Models from FDA and WHO

Organization	Purpose of Innovation Model	Principles/Tenets
**FDA Collaboration Phase Playbook**	The Collaboration Playbook was developed to support the Innovation Pathways programme, sponsored by the FDA, in an attempt to simplify and streamline the way that innovators work with the agency. This collaboration model is used to facilitate a more interactive and fluid product development model during the early stages of clinical innovation between the FDA and innovators. As stated on the FDA website, the guiding principles behind the Collaboration Phase include creating a shared understanding of product success including its benefits and risks, creating solutions that facilitate forward progress, allowing experimentation, prototyping, learning, and striving for greater transparency.	**Principle 1:** Share an In-Depth, Common Understanding of Success **Principle 2:** Apply Best Practices in Framing Benefit and Risk **Principle 3:** Create Solutions that Facilitate Forward Progress **Principle 4:** Improvise, Experiment, Prototype, Test, and Learn **Principle 5:** Full Transparency in Decision Making
**WHO Health “3D” Innovation Cycle**	The 3D Innovation Cycle represents a schema that applies principally to developed countries and the diseases which predominantly affect them, where effective demand and the population's health needs most closely coincide. For conditions such as cancer and asthma, incremental improvements are commonplace, and companies have a reasonable assurance that healthcare providers and patients will purchase their products. That provides the basic economic and financial incentive for innovation. Whatever the various problems encountered in the innovation cycle, either technical or in terms of the policy framework, it broadly works for the developed world and sustains biomedical innovation directed at the improvement of public health. (Excerpt from the 2006 WHO report Public Health Innovation and Intellectual Property Rights: Report of the Commission on Intellectual Property, Property Rights, Innovation and Public Health.)	The 3D Innovation Cycle is an iterative framework that consists of the following collaborative efforts for clinical innovation: **Discovery:** ◯ Lead Identification/Optimization ◯ Basic Research **Translational Research** **Development:** ◯ New/Improved Tools ◯ Preclinical and Clinical Development **Market Approval and Manufacture** **Delivery** ◯ Getting Products to Patients

The IFPMA's PIP model is composed of healthcare delivery characteristics that are necessary for an effective clinical innovation environment to grow locally, nationally and internationally [9]. Table 2 lists the attributes that the IFPMA outlines in its PIP model [9].

The model goes into greater detail regarding the tactical aspects of each of these areas. We saw in our research congruent themes between IFPMA, WHO and FDA in this regard, pertaining to scalable clinical innovation models for LMICs and LICs to use as their strategic compass in navigating towards locally sustainable research efforts. Table 3 is a summary of both the FDA's and WHO's adaptable innovation models.

The model ultimately enables LMIC and LIC innovators to transition from being the primary recipients of innovation, to originators of clinical innovation. It will also take bold and unconventional thinking in order to overcome the socioeconomic and infrastructure barriers that innovators face within these regions of the world. Both WHO and the FDA provided solid narrative guidance on implementation approaches to their models. However, there was limited evidence, beyond their illustrative examples, of the measurable outcomes of the use of these models by LMICs and LICs in our systematic review. This has inspired our thinking as healthcare innovators, to re-architect and integrate best practices from IFPMA, WHO, the FDA, and the new agile Open Source technology and Open Science frameworks, which are gaining greater acceptance as a platform of choice for clinical intelligence communities, as the next generation of collaborative research [10].

## 5. Next Generation of LMIC and LIC Virtual Collaboration Models

The movement towards Open Source-Open Science collaboration is evident with the new e-health platforms that are taking shape to address these challenges. As the Global Health community enters into a broader range of eHealth adoption efforts, we see pioneering growth and expansion of mHealth (mobile phone technology used for healthcare data exchange and patient engagement) solutions that are taking a progressive approach to enabling more effective patient engagement and peer-to-peer clinical decision support at the point of care. This has also led to supporting new patient engagement research models for public health bio-surveillance and education efforts related to infectious disease prevention and control and medication adherence in LIC and LMIC regions of the world. As a result, some mHealth initiatives have moved the needle with the adoption of new patient engagement efforts, simply due to leveraging this new technology. Yet it is still in its infancy as a stand-alone solution that ignites locally based clinical innovation. The impact and sustainability of clinical innovation powered by mHealth solutions are still uncertain, due to the following factors outlined by WHO in its 2015 guide for mHealth solutions [11], shown in Table 4 below:

**Table 4.** Factors that Prohibit Growth of mHealth Solutions for Global Health Innovation

WHO Factors that Impede mHealth Adoption
• Conflicting healthcare priorities
• Unsustainable operating costs
• Inability to consistently measure clinical and cost effectiveness
• Lack of harmonized healthcare policy and governance models to support mHealth/telehealth initiatives
• Lack of knowledge concerning the possible application of mHealth and public health outcomes
• Lack of IT infrastructure to support mHealth and telehealth programmes
• Patient literacy, privacy and cultural issues

The goal of an integrated Open Source-Open Science platform is to take mHealth one step further, by enabling re-usable clinical intelligence that can be shared and redistributed in the context of clinical innovation before, during and after care is delivered. As a result, mHealth thus becomes an essential building block to this framework by providing a timely data feed for the innovation process. When mHealth is coupled with an Open Source-Open Science virtual collaboration environment, it will enable LMIC and LIC research scientists to engage in interactive drug discovery and global knowledge sharing for clinical innovation. As a result, it may reduce drug discovery lag time, by enabling timely collaborative clinical trial data sharing and bio-surveillance intelligence across all phases of drug discovery.

## 6. Conceptual Open Source-Open Science System Design Approach

Our conceptual Open Source-Open Science model, Project Orchid, incorporates the above requirements, in order to strengthen existing clinical trial partnerships and support new collaborative efforts that have disparate geographic, cultural and regulatory drivers. We have designed an integrated operational governance and clinical innovation engagement platform that can be adapted to the needs of each stakeholder organization. The intent of our model is to illustrate, through a multi-national TB clinical trial cohort, the potential outcomes of using the Open Source-Open Science framework (Table 5).

## 7. Open Source-Open Science Innovation Framework

**Figure 1.**
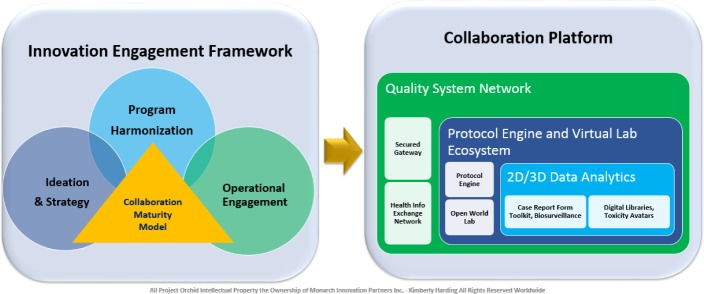
Project Orchid Open Source-Open Science Innovation Framework

**Figure 2.**
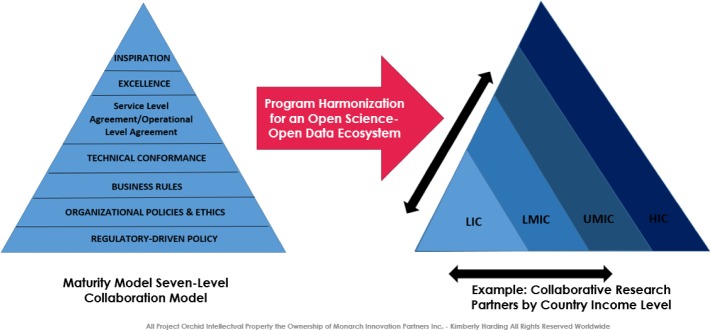
Project Orchid Collaboration Maturity Model

Project Orchid consists of two integrated offerings: **An Innovation Engagement Framework** and a technology-enabled **Collaboration Platform** (Figures 1 and 2), designed to address disparate institutional and cultural barriers that impede medical innovation, by providing Clinical Research Organizations, government-sponsored public health and research agencies, academia, nongovernment/non-profit agencies and pharmaceutical and medical device companies with an interactive drug discovery environment and best practices framework, to harmonize their clinical trial and public health programme governance approaches to produce improved research and care delivery outcomes.

**Table 5.** Open Source-Open Science Clinical Trial Platform - Multi-National TB Drug Discovery Strategy

Collaborative Drug Discovery Strategy & Outcomes	
**Goals**	Establish an Open World Clinical Trial Collaboration Metaverse environment to facilitate Toxicity and Efficacy data sharing and Bio-surveillance across Clinical Trial Genomic Avatars Host a scalable technology platform for Adaptive Clinical trial frameworks
**Challenges**	Language Barriers Clinical practice and operational governance differences across countries Lack of integrated Health Information Exchange Networks and/or gateways outside of native Health IT/Information Management ecosystem for cross-trial data sharing and analysis Lack of harmonized Clinical Imaging Trial data management and workflow approaches Clinical practice and operational differences, which limit innovation opportunities outside of organization
**Expected Outcomes:**	Enable accelerated data harvesting and optimized bio-surveillance of potential serious adverse events across multiple genomic profiles that meet Phase II/Phase III data monitoring requirements for ICH and FDA investigational new drugs/vaccines An established Open Science Knowledge Network for cross-trial data analysis within a defined research community
**Shared Systems:**	Project Orchid Platform mHealth (mobile telecommunication) Platform
**Clinical Trial Quality System Management Regulatory Frameworks:**	ICH Technical Specifications for Harmonization The three National and Regional Clinical Trial Regulatory Frameworks

## 8. Open Science Collaboration Maturity Model

Our proposed **Open Science Collaboration Maturity Model** is used to identify and address current process, resource and competency gaps and disparities in key areas that impede medical innovation across the group of stakeholder organizations. This exercise will enable innovation teams to apply knowledge management, process harmonization and re-engineering techniques and risk mitigation strategies to bridge potential points of failure within the collaboration effort. The model is a process performance hierarchy, designed to address peer-to-peer organizational disruption, due to change management stressors that new medical innovation partnerships experience as an outcome of joint programme initiatives with financial and regulatory considerations. The focus of the model is designed for both healthcare solution development (i.e., Population Health initiatives for Public Health programmes) and drug discovery efforts, across national and multi-national engagements. Project Orchid's Model is based on three integrated aspects of collaboration:

- **Programme and Policy Harmonization:** This consists of addressing regulatory and cross-cultural organizational dynamics, process adoption and motivation models.
- **Ideation and Strategy:** This consists of addressing medical innovation modelling and clinical trial bridging strategies.
- **Operational Engagement:** This consists of facilitating, across the cohorts, the identification of service-level key performance indicators, knowledge management and delivery system optimization efforts after the launch of their medical innovation.

The structure of the model is to encourage transparency and process agility, while leveraging the collective strengths of all stakeholder organizations in order to accelerate opportunities for business development, product innovation and service management in a more fluid manner. This framework aligns to the ICH Quality System concept tenets: Process Performance and Product Quality Monitoring System Corrective Action/Preventive Action (CA/PA); System Change Management; System Management Review; Knowledge Management and Quality Risk Management [5, 6]. Each level of the maturity model corresponds to a key set of measures and methods that a collaborative partnership focuses on in its efforts to enable sustainable innovation. A facilitated Strengths, Weaknesses, Opportunities and Threats (SWOT) analysis is done across all core stakeholders of the partnership to determine how to address disparities in the “as-is” and “to-be” model for collaboration, followed by a risk mitigation and change management governance model adopted at all levels of the joint venture. The structure of the model is to encourage transparency and process agility, while leveraging the collective strengths of all stakeholder organizations in order to accelerate opportunities for business development, product innovation and service management in a more fluid manner [10–13].

It is recommended that, prior to initiating a formal collaborative partnership, an organization should perform a self-assessment to be fully aware of its SWOT findings and perform an internal cognitive walkthrough, or “what if” scenario with potential partnerships, in preparation for change management stressors or business drivers that may be encountered as part of the harmonization effort for joint ventures [12, 13]. The table below (Table 6) itemizes each level of the maturity model and how it is applied across all three domains of the collaboration framework.

## 9. Clinical Trial Data Quality and Regulatory Policy Harmonization

The incremental nature of the Collaboration Maturity Model can be applied when multi-national research teams with LIC and LMIC partners attempt to harmonize the following GCP frameworks and regulatory standards that align to key bioethics and privacy guidelines related to the Clinical Trial Data Management system used in the Life Science industry. They include, at a minimum, the standards listed in Table 7:

## 10. Open Source-Open Science Solution Architecture and System Design

A proposed Open Source-Open Science Collaboration solution architecture should leverage Open Source technology frameworks for 2D and 3D data visualization and healthcare interoperability standards, such as HL7 Fast Healthcare Interoperability Resource (FHIR), Clinical Data Architecture (CDA) and Digital Imaging and Communication in Medicine (DICOM) standards, which are widely used in electronic medical records, medical devices, picture archiving systems and registries worldwide, to enable system-to-system integration and data exchange across the clinical research and care management continuum [13–20]. Open Source-Open Science platforms, like Project Orchid, will also leverage technology components, such as Linux and Drupal, and long-term scalable telecommunication frameworks, such as Internet2 and Unified Communication protocols, which optimize secured clinical data exchange in low bandwidth regions of the world. The proposed implementation model for Project Orchid is to incorporate these standards (Table 8) within Project Orchid's capabilities as a shared Platform-as-a-Service offering, to enable global pharmaceutical and biotechnology firms and government-based Life Science agencies to bridge the clinical data exchange connectivity divide with their LIC and LMIC pilot sites, which have very limited IT and mHealth infrastructure resources.

## 11. Unified Communication and Usability Standards

The following table (Table 9) is a partial list of the protocols and standards that will be used in the development of the Unified Communications recommended by Texas A&M Internet2 Technology and Evaluation Center (ITEC).

We will also use the ISO 9241-11 standard to verify the following criteria for user interface design, recommended by Virginia Tech's Advanced Research and Computing, Visionarium Lab: effectiveness, efficiency and satisfaction (ISO 1998). This will take into account both the visually and hearing impaired as well. The use of these standards will ensure effective adoption across diverse organizational, language and cultural attributes for our proposed multi-national pilot programme.

**Table 6.** Project Orchid Collaboration Maturity Model Reference Table

Collaboration Level	Measure Attributes for Level	Method for Assessing Harmonization for Level	Application to Collaboration Domains	Timing in Engagement Lifecycle
**Policy**	Local, Regional, National, International Regulatory Standards and Guidelines	Self-audit and programme-level audit exercises per the specifications of the regulation(s)	Programme and Policy Harmonization Ideation and Strategy Operational Engagement	Recurring, per the specifications of the regulation(s)
**Organizational Ethics**	Business Value Statements and Public Policy Guidelines	Self-audit and programme-level audit exercises per the specifications of the adopted set of organizational ethics	Programme and Policy Harmonization Ideation and Strategy Operational Engagement	Recurring, per the specifications of the adopted set of organizational ethics
**Business Rules**	Standard Operating Procedures, Workflows and Protocols	Workflow re-engineering and synchronization across stakeholders, resulting in measurable process optimization	Programme and Policy Harmonization Operational Engagement	Key role-based performance efforts across each business domain of the programme
**Technology Conformance**	Infrastructure, Security and Data Architectures, System-to-System integration and process automation, Inbound and Outbound Connectivity Channels	Vendor attestation for technical conformance via interoperability assessments and the use of Open Source tools	Programme and Policy Harmonization Ideation and Strategy Operational Engagement	Ideation and Strategy, Operational Engagement phases of the initiative
**Service Level Agreements/Operational Level Agreements**	Business, Operations and System-level Performance Agreements	Customer engagement key performance indicators (KPIs) and system-level non-functional requirements that directly impact business and system-level operational performance	Operational Engagement	Project Initiation and revisited on a recurring basis in Operational engagement through service management efforts
**Excellence**	Continuous improvement targets for business and system-level key performance indicators	Targeted customer engagement KPIs and system-level non-functional requirements that have been designated as market differentiators in overall performance improvement for the initiative	Ideation and Strategy Operational Engagement	Project Initiation and revisited on a recurring basis in Operational engagement through service management efforts
**Inspiration**	The intrinsic reward of achieving the goals of partnership and its impact on the recipients of the product/service and industry as a whole	Revisiting the mission and vision statements of each stakeholder, highlighting the ideals they promote and incorporating them within the culture of the initiative	Programme and Policy Harmonization Ideation and Strategy Operational Engagement	Throughout the course of the partnership as a method of team building and fostering trust across organizational and geographical boundaries

## 12. Open Science User Profiles

In order to ensure that each member of the initiative has the appropriate access rights to the Open Source-Open Science Platform, the system will provide role-based capabilities to maintain the data integrity of the system and ensure alignment to Good Clinical Practices (GCP). Table 10 is a limited representation of a typical user profile configuration that a Clinical Research Organization (CRO) may collaborate with.

## 13. Implementation Model for an Open Source-Open Science Initiative

In order to illustrate how an Open Source-Open Science platform can be implemented for a viable clinical innovation effort across multi-national stakeholders, Project Orchid has targeted two scenarios: a multi-national Phase III TB Clinical Trial for Drug Toxicity/Efficacy and Bio-surveillance; and a supporting Open Science Knowledge Network to disseminate clinical intelligence to a specific TB research collaboration community. Our goal is to pilot this concept with our research partners.

**Table 7.** Project Orchid Clinical Trial and Data Management Standards

International-Based Clinical Data Quality Standards and Guidelines	US-Based Clinical Quality Data Standards and Guidelines
ICD-10 ICH (International Council for Harmonisation) Guidance Documents for Good Clinical Practices ICH Common Technical Document (CTD) ICH MedDRA EU Directive 2001/20/EC EU Directive 2005/28/EC World Medical Association Declaration of Helsinki	Dept. of Health and Human Services Agency for Healthcare, Research and Quality Registry Standards and Guidelines Dept. of Health and Human Services FDA Code of Federal Regulations Part II Electronic Signatures and Records Dept. of Health and Human Services FDA Computerized System Used in Clinical Trials - Good Clinical Data Management Practices, version 4 (CDISC) Clinical Data Interchange Standards Consortium, Operational Data model (ODM) (CONSORT) Consolidated Standards of Reporting Trials documentation Dept. of Human Services Health Insurance Portability and Accountability Act (HIPAA) Personal Health Information (PHI) regulations for covered entities and business associates and Federal Patient's Rights policies for Opt-In/Opt-Out Data Sharing events Society for Clinical Data Management Guidelines for clinical trial quality management systems

**Table 8.** Project Orchid Open Source Technology, Data Architecture and Health Interoperability Frameworks

Web and Telecom Infrastructure	Data Architecture & Governance Frameworks	Open Source Software Architecture Standards	Healthcare Interoperability and IT Quality Standards	Health Technology Assessment and Service Management Frameworks
Cloud Computing Wireless Telecom Web2.0/3D Open Source Server Solutions HTML 5	NIH Common Data Element NIH - Agency for Healthcare Quality and Research Patient Registry HealthIT.gov Quality Data Model Schema-less and Dynamic Data Architecture Frameworks	Drupal OpenGL X3DOM X3D The Open Group Architecture Framework v 9.1	HL7 FHIR, CDA R2 DICOM for Radiology IHE Quality Profiles HealthIT.gov S&I Framework ISO 9241-11 My Blue Button	WHO Health Technology Assessment for Medical Devices European Network for Health Technology Assessment – The HTA Core Model

**Table 9.** Project Orchid Unified Communication Protocols

Standard Unified Communication Protocols	Description
IETF RFC 3261 – Session Initiation Protocol (SIP)	• This is the basis of all current voice and video communications globally. It is used for establishing communications channels. There are at least 40 other RFCs associated with SIP that are used for various aspects of the call setup
IETF RFC 3428 SIMPLE Protocol	• This standard supports Instant Messaging over SIP
IETF RFC 5139 Presence Information Data Format Location Object (PIDF-LO)	• This is the industry standard format for embedding location information into a message
IETF RFC 4566 Session Description Protocol	• This standard allows end users to connect via voice, video, text or other media
OASIS Common Alerting Protocol (CAP)	• This is the standard that allows the transmission of emergency alert mass notifications

## 14. Multi-National TB Phase II/III Clinical Trial Toxicity and Efficacy 3D Bio-surveillance

The scope of the proof of concept and pilot is to build, test and deploy, in the field, an Open World Clinical Trial Collaboration Metaverse ecosystem between several central and sub-Saharan African countries, India, and the US, to facilitate a TB Vaccine Toxicity and Efficacy cross-trial data sharing initiative. The technology platform will simulate a vaccine development initiative across these locations with a shared virtual collaboration network that has a 2D/3D Genomic Toxicity and Efficacy Avatar environment, to detect and monitor toxicity and efficacy outcomes of specific formularies in Phase II/III TB clinical trials. The Human Avatar will display growing or diminishing contraindications that are reported via Case Report Forms from the participating researchers, as well as other relevant data feeds, such as laboratory information systems, health information systems, or radiological imaging systems, with projected toxicity or efficacy modelled by the current drug formulary under investigation. These results will be viewable on a toxicity and efficacy intelligence dashboard and a three-dimensional heat map of the areas affected on the Avatar. For example, targeted organs, such as the kidneys or liver, can be highlighted and “virtually scanned” for image manipulation and shared across the clinical research community for analysis. We will also include Open Source and DICOM viewers for 2D and 3D radiology images and pathology samples from submitted patient-level data sets for peer-to-peer consultations and retrospective clinical analysis on short- and long-term therapeutic outcomes.

**Table 10.** Project Orchid Open Science User Categories

Clinical Innovation User Profiles
Principal/Chief Investigator
CRO/Site Level Clinical Investigator
Clinical Trial Quality System Lead/Engineer
Investigational Review Board Member
Data Monitoring Members, Research Librarians and Healthcare Data Stewards
Pharma/Biotech CRO sponsors
Site Level Research Scientists and Epidemiologists
IT and Telecommunication System Administrators
Clinician and Healthcare Workers
Clinical and Public Health Regulatory Grantor Representatives

The results will also be fed into a Cross-Trial Drug Toxicity and Efficacy Registry for concurrent and systematic review by regulatory bodies, the sponsoring IRB, and Data Monitoring committees within the research community. The following organizations have confirmed their interest in participating in usability testing and piloting this Project Orchid scenario:

- D-Tree International: An NGO that specializes in Public Health programmes and mHealth solutions in sub-Saharan Africa and India
- Handheld Solutions and Research Labs (HANDSREL): HANDSREL is a company based in Bangalore, India, which develops and provides mobile data collection solutions and services
- ITOCA (Information Training and Outreach Centre for Africa): ITOCA is a South Africa-based NGO training, research and outreach centre for clinical research and academic institutions in Africa and their partners
- Muhimbili University of Health and Allied Sciences, Dar es Salaam, Tanzania
- Central Africa Network on TB, HIV/AIDS and Malaria: A consortium of the University of Tubingen, Germany, and eight clinical research institutions in the Republic of the Congo, Gabon and Cameroon
- Programme of Health of Reproduction and Family, an NGO from the Democratic Republic of Congo
- Virginia Tech Advanced Research Computing-Information Technology and Visionarium Lab
- Texas A&M Internet2 Technology Evaluation Center
- University of Maryland College of Information Studies

**Figure 3.**
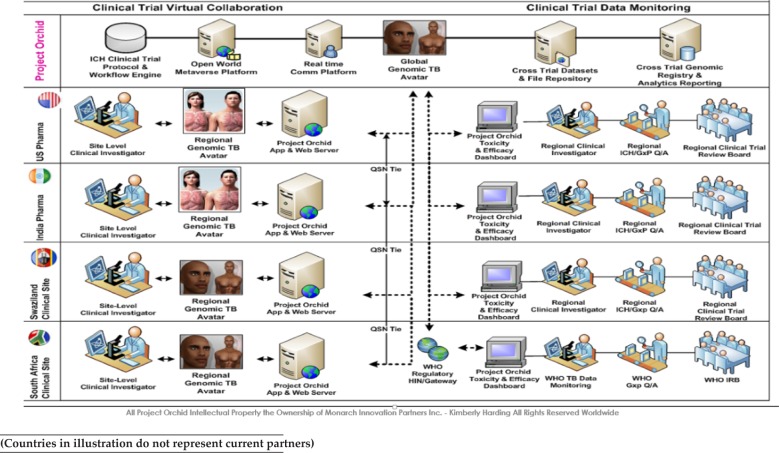
Project Orchid Clinical Trial Innovation Platform for Infectious Disease Drug Discovery

**Figure 4.**
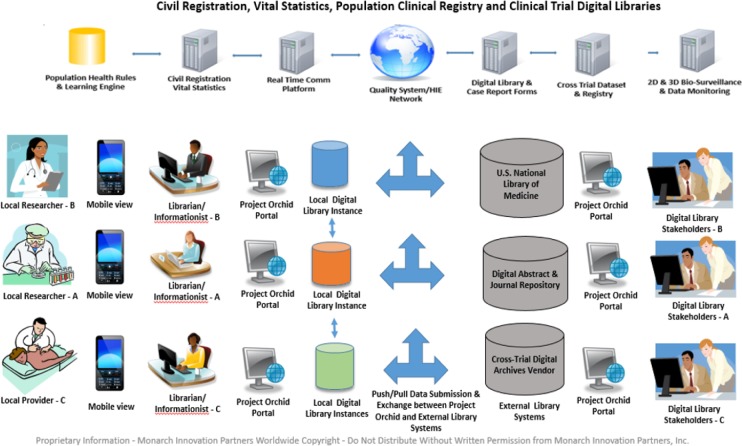
Project Orchid Open Science Digital Library and Knowledge Network

Below are diagrams (Figures 3 and 4) that illustrate the Project Orchid conceptual collaboration platform.

## 15. Conclusion

The current solutions in the LIC and LMIC clinical innovation domain are primarily third party vendor systems, which require unsustainable IT infrastructure and software management support, or heavily paper-based processes supplemented with standard IT desktop applications and databases. There are also very limited Open Source-Open Science, health information exchange and mHealth networks that are integrated into multi-national cohorts and these do not address the dynamic clinical trial frameworks used in drug discovery. Collectively, these systems do not enable the robust capabilities needed to sustain near-real time clinical innovation or meet the regulatory standards for compliance to GCP systems for clinical trial data management. As a result, LICs and LMICs cannot transition effectively from manual administrative efforts in order to participate in multi-national clinical trials and public health innovation, which directly impedes their ability to mature as an organization.

As a Global Health community, we must advocate for both agnostic and agile technology architecture frameworks that enable technical, syntactical and semantic interoperability and business process harmonization across LIC and LMIC cohorts and their MIC and HIC stakeholders, for true clinical innovation progress worldwide. Open Source and Open Science Innovation frameworks are leading this incremental transformation in our Life Science domain. This movement has become our next wave of healthcare modernization and disruption, in bridging the digital divide within our Global Health community.

This is also driving the development of new health IT solutions around the world. From our observation and implementation experience, the most effective disruptive technology solutions within the Global Health domain for clinical innovation will need to provide scalable Open Science-Open Source life science solutions, specifically designed and priced to meet the data sharing challenges that LIC and LMIC clinical research and public health organizations face in clinical trial and medication innovation efforts in a global market. This entails developing re-usable and extensible technology that can integrate with local and international health information exchange networks that support data liquidity between proprietary and Open Source electronic medical record systems, laboratory and radiology systems and mHealth platforms, which are on a par with MICs and HICs. In addition, they will need to provide virtual Open Science collaboration environments, which enable access to near-real time clinical intelligence, science breakthroughs and new drug discovery partnerships that can accelerate the business development capabilities of LMICs and LICs in the Life Science industry. An example of this is the 2D/3D Open World Metaverse and Digital Library we are developing within our proof of concept.

The opportunity for innovation empowerment is within reach for emerging markets and LMICs, when global ingenuity meets Open Source-Open Science technology.

## 16. Conflict of Interest

The author declare no conflicts of interest.
